# Digitally Guided Augmentation With an Autogenous Bone Cylinder—Digital CarroTrack Technique: A Case Report

**DOI:** 10.1155/crid/1545076

**Published:** 2026-07-31

**Authors:** Jakub Kwiatek, Sylwia Pokorska, Marta Leśna, Noemi Konopelski, Oskar Barczak, Marcin Lenkowski, Ilona Różewicz, Paulina Łojewska-Pabiś

**Affiliations:** ^1^ Kwiatek Dental Clinic, Poznań, Poland

**Keywords:** autogenous bone graft, bone augmentation, carrot technique, dental implants, Digital CarroTrack Technique, digital implant planning, guided implant surgery, Khoury technique, surgical guide

## Abstract

This case report presents the surgical and prosthetic treatment of posterior mandibular tooth loss using a digitally guided augmentation technique with an autogenous bone cylinder, referred to as the Digital CarroTrack Technique. The method represents a digital modification of the autogenous bone cylinder harvesting technique (carrot technique) described by Khoury and integrates implant planning with guided bone augmentation. A 33‐year‐old female patient presented for implant‐prosthetic rehabilitation of missing mandibular molars following previous orthodontic treatment. Diagnostic assessment included CBCT imaging, intraoral scanning, and clinical examination. Implant placement at Sites #36 and #46 was digitally planned using dedicated software, and a surgical guide incorporating sleeves for implant drills, trephine drills, and stabilizing screw placement was designed. Bone cylinders were harvested using a trephine guided through the custom‐designed sleeve and stabilized laterally to the implant sites with fixation screws, followed by augmentation with a bone substitute material. After a 3‐month healing period, digital impressions were obtained, and screw‐retained zirconia crowns on Ti‐base abutments were fabricated and delivered. Radiographic and clinical evaluation confirmed successful bone reconstruction and stable peri‐implant tissues. Stabilizing screws were removed during a follow‐up visit using a minimally invasive approach. Three‐year follow‐up demonstrated stable hard and soft tissues and satisfactory functional and aesthetic outcomes. The presented technique enables precise integration of digital implant planning with localized autogenous bone augmentation and may represent a minimally invasive approach for the reconstruction of small alveolar ridge deficiencies in implant dentistry.

## 1. Introduction

Replacement of missing teeth following previous extractions represents one of the fundamental surgical procedures in oral surgery. Following tooth loss, progressive resorption of both bone and soft tissues occurs at the site of the edentulous ridge. Studies have shown that within the first 6 months after extraction, the alveolar ridge may lose approximately 29%–63% of its width and 11%–22% of its height, with the most pronounced changes occurring during the first 3–6 months of healing [1].

One of the teeth most frequently requiring replacement in young patients is the first permanent molar. These teeth belong to the group of nonsuccedaneous teeth, as they develop from the distal extension of the primary dental lamina, from which the primary (deciduous) teeth also originate [2]. They typically erupt around the age of 6 and are the first permanent teeth to appear in the oral cavity. Their early eruption in the dentition, combined with difficulties in maintaining proper oral hygiene during childhood, predisposes them to the development of dental caries and premature loss [3].

In many cases, advanced carious lesions or complications associated with endodontic treatment result in tooth extraction being the only viable treatment option.

Immediate implant placement is increasingly recommended, as it helps limit alveolar ridge resorption and preserve the architecture of hard and soft tissues at the postextraction site [4]. However, immediate implant placement is not always feasible, among other reasons due to the patient′s age, local anatomical conditions, the presence of inflammation, or the fact that implant therapy was not planned at the time of extraction.

In the absence of immediate implant placement, progressive changes occur in the alveolar ridge over time. Bone resorption primarily takes place in the horizontal (buccolingual) dimension, with the most pronounced changes observed during the first months following tooth extraction [5]. In the maxilla, tooth loss may additionally lead to progressive pneumatization of the maxillary sinus [6], combined with the reduction in alveolar ridge height; this often necessitates additional augmentation procedures prior to implant therapy [7].

Along with the loss of hard tissues, a reduction in soft tissues also occurs, which may lead to deterioration of the emergence profile of the future prosthetic crown, food impaction, and difficulties in maintaining proper oral hygiene [8].

To restore proper function and aesthetics, standard treatment often requires augmentation of the hard tissues followed by implant placement after an appropriate healing period. The next stage of treatment involves implant exposure combined with soft tissue augmentation to achieve an appropriate emergence profile.

This treatment approach is typically time‐consuming, requires multiple visits, and involves several surgical procedures, even though it concerns the restoration of a single tooth.

Modern dentistry, based on digital technologies and the ability to plan the entire procedure with consideration of the implant and prosthetic crown positions, enables a reduction in both the number of surgical procedures and the overall treatment time [9]. An important aspect is also the predictability and safety of the procedure itself. The use of surgical guides enables more controlled implant positioning in accordance with the prosthetic plan and reduces the risk of damage to adjacent teeth and important anatomical structures [10].

A remaining challenge is the ability to plan bone augmentation necessary to provide adequate support for the soft tissues, which allows for achieving a predictable aesthetic and functional outcome. Preoperative planning of such a procedure constitutes an important component of implant therapy.

One of the augmentation methods using autogenous bone is a bone graft performed with the use of the “Carrot Technique” described by Khoury and Doliveux [11]. This technique involves harvesting a cylindrical bone graft from the future implant site using a dedicated bone trephine and subsequently stabilizing it with osteosynthesis screws laterally to the planned implant position in order to reconstruct the missing bone volume.

From the perspective of healing time and patient comfort, the use of an autogenous bone core allows the need for a separate donor site procedure to be avoided, as both the harvesting of the graft and its placement are performed within the same surgical field.

The use of autogenous material provides high biological potential. Autogenous bone exhibits osteogenic, osteoinductive, and osteoconductive properties, which is why it is considered the “gold standard” in bone augmentation in implantology [12].

In the traditional “Carrot Technique,” performed without navigation using a surgical guide, harvesting the bone cylinder should be carried out in a manner that simultaneously determines the correct position of the future implant. In cases of a thin alveolar ridge or proximity to important anatomical structures, this procedure may be technically demanding. Additional difficulty may also arise from the stabilization of the harvested bone core at the defect site.

A particular example is the mandible in the region from the first premolar to the first molar, where the mental foramen is located in close proximity to the surgical field. The mental foramen contains the mental nerve, which is the terminal branch of the inferior alveolar nerve. Under such conditions, there is a risk of injury to neural structures during preparation of the implant site or harvesting of the bone graft [13, 14].

The position of the mental foramen shows considerable anatomical variability. In most cases, it is located between the first and second mandibular premolars (approximately 56%), whereas in about 36% of cases it is found directly in the region of the second premolar [15].

Therefore, the placement of stabilizing screws in this region may be associated with a risk of injury to neural structures.

The placement of a stabilizing screw requires careful selection of its angulation, length, and position to ensure adequate stabilization of the bone core, avoid injury to anatomical structures, and not interfere with proper implant placement.

A possible solution to these challenges is the use of a specially designed guiding sleeve for the trephine drill and an additional guiding sleeve for the drill used to place the stabilizing screw.

This case report presents the application of this approach, referred to by the authors as the Digital CarroTrack Technique, which represents a digital modification of the carrot technique, including the design phase and long‐term follow‐up.

This solution enables a more controlled execution of the procedure in areas adjacent to anatomically and clinically significant structures.

## 2. Case Presentation

A 33‐year‐old female patient presented to Kwiatek Dental Clinic for the replacement of missing molar teeth. The patient had previously completed orthodontic treatment performed at another dental office several years earlier. As the patient did not consent to further orthodontic treatment aimed at correcting the midline and improving occlusal conditions on the left side, a decision was made to plan implant therapy. The patient was generally healthy, with no systemic diseases and no contraindications to implant surgery.

As part of the diagnostic protocol, cone‐beam computed tomography (CBCT) was performed (Orthophos SL 3D, Dentsply Sirona, Bensheim, Germany), along with intraoral scans (TRIOS, 3Shape, Copenhagen, Denmark), and intraoral photographic documentation. A comprehensive clinical examination, including both subjective and objective assessment, was also conducted.

Clinical examination revealed a deficiency of alveolar bone volume in the posterior mandibular region, which limited the possibility of standard implant placement. Radiological assessment using CBCT confirmed insufficient horizontal width of the mandibular alveolar ridge.

**Figure 1:**
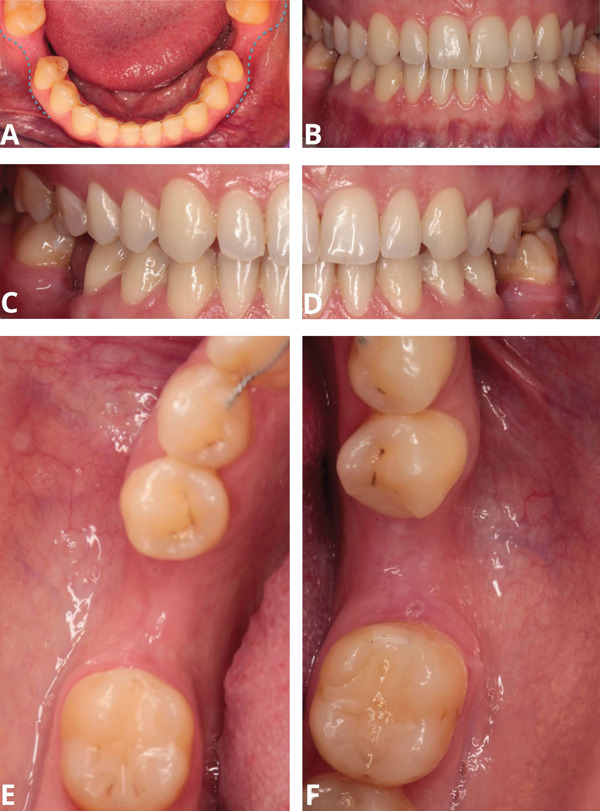
Intraoral situation before the initiation of implant therapy. (A) Occlusal view of the mandibular arch, (B) frontal view in occlusion, (C) right lateral view in occlusion, (D) left lateral view in occlusion, (E) occlusal view of the left posterior mandibular region at the planned implant sites, and (F) occlusal view of the right posterior mandibular region at the planned implant sites.

**Figure 2:**
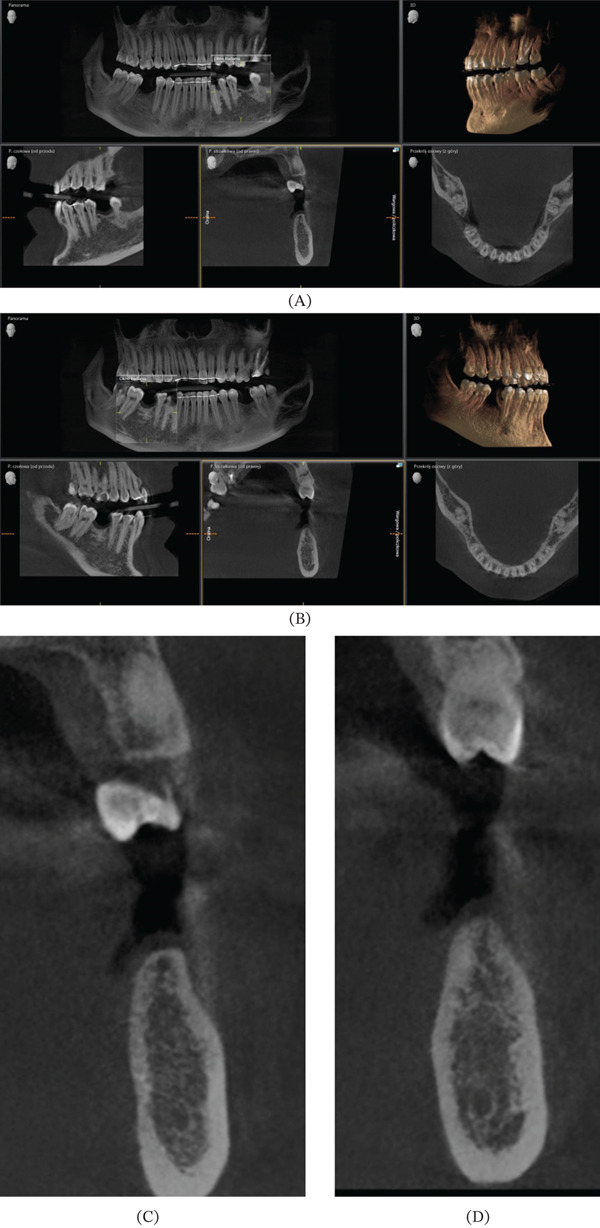
Radiological diagnostics prior to treatment based on CBCT examination. (A) CBCT evaluation of the left posterior mandibular region, (B) CBCT evaluation of the right posterior mandibular region, (C) cross‐section—left posterior mandible, and (D) cross‐section—right posterior mandible.

The intraoral situation and occlusal relationships are presented in the figures below (Figures 1A–F and 2A–D). The sequence of treatment stages is summarized in Table 1.

**Table 1:** Treatment stages and procedures.

Treatment stage	Description
Initial visit	Patient consultation, clinical examination, and CBCT imaging
Treatment planning	Digital implant planning and preparation of surgical guide
Surgical procedure	Implant placement with autogenous bone cylinder augmentation
Healing phase	Osseointegration period
Prosthetic rehabilitation	Implant exposure and final prosthetic restoration
Follow‐up	Long‐term clinical and radiological evaluation

Implant‐prosthetic treatment was planned, and the subsequent stages of the procedure were performed according to the Digital CarroTrack Technique protocol.

The surgical planning began with determining the implant positions (MIS C1, MIS Implants Technologies Ltd., Bar‐Lev, Israel) in Sites 36 and 46 using Zirkonzahn software (Zirkonzahn GmbH, Gais, Italy).

The implants were planned in an appropriate position relative to the soft tissues already at the design stage, with their placement deepened subcrestally by 4.5 mm in relation to the crest of the alveolar ridge and the thickness of the soft tissues.

**Figure 3:**
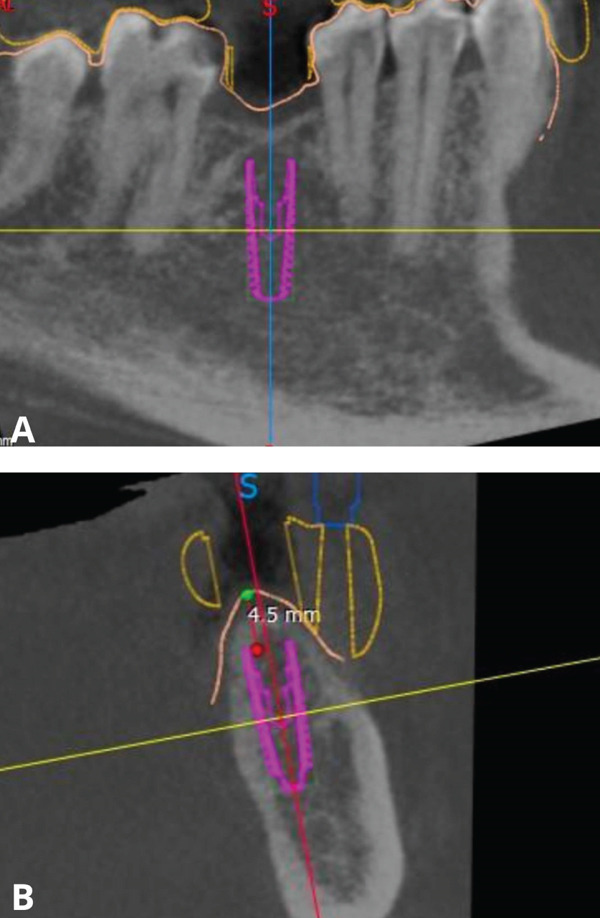
Implant placement planning based on CBCT. (A) Coronal view and (B) sagittal view.

This planning was performed in accordance with the “zero bone loss” concept described by Linkevičius et al. [16], which assumes deeper subcrestal implant placement in cases of insufficient soft tissue thickness. In the present case, the soft tissue thickness was approximately 1.5 mm; therefore, the implant was positioned to achieve a target soft tissue height of approximately 4 mm (Figure 3A,B).

The next stage of the design process involved planning the position of titanium osteosynthesis screws, 6 and 8 mm in length (Surgident, Korea; distributed by 3Z), which were used to stabilize the bone core and ensure an adequate volume of hard tissue in the implant area. The distance between the screw and the buccal bone surface was set at 2.9 mm, allowing optimal compression and stabilization of the grafted bone core.

During the design process, components of a surgical guide positioning system were utilized. An important element of this system is a drill equipped with a depth stop, enabling safe insertion of stabilizing pins while maintaining an adequate distance from the implants and adjacent anatomical structures.

**Figure 4:**
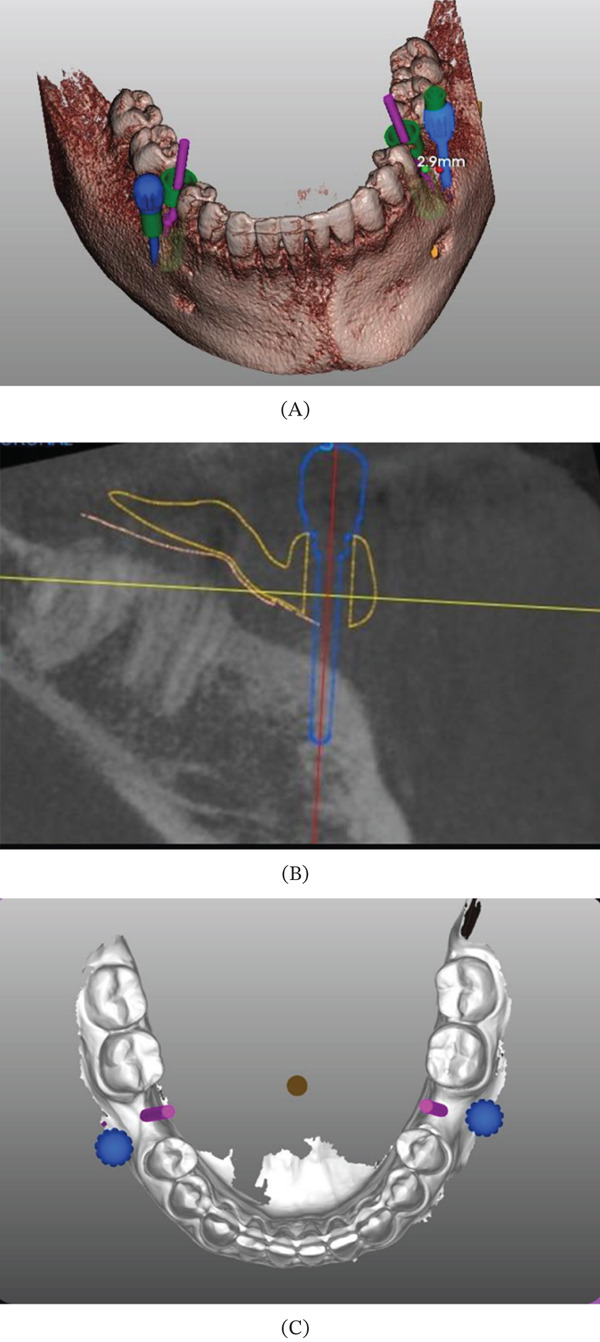
Planning of stabilizing screw positions in the digital design. (A) Three‐dimensional visualization of the mandible with planned implant and stabilizing screw positions, showing the planned 2.9‐mm distance from the buccal bone surface, (B) CBCT cross‐sectional image illustrating the relationship between the planned stabilizing screw and the anatomical structures of the mandible, and (C) occlusal view of the design with the positions of the stabilizing screws marked.

The narrow guiding sleeve used in the design allows the use of stabilizing screws with a diameter of 1.2–1.4 mm (Figure 4A–C).

After verifying the implant positions in the 3D planning environment, a surgical guide was generated containing guiding sleeves for implant placement (standard MGUIDE system sleeves, MIS Implants Technologies Ltd., Bar‐Lev, Israel), dedicated sleeves for the trephine system developed by the authors of the present study and covered by Patent Application No. PL 449269, as well as sleeves for the drill used for the insertion of stabilizing screws based on the standard pinning sleeves of the MGUIDE system.

**Figure 5:**
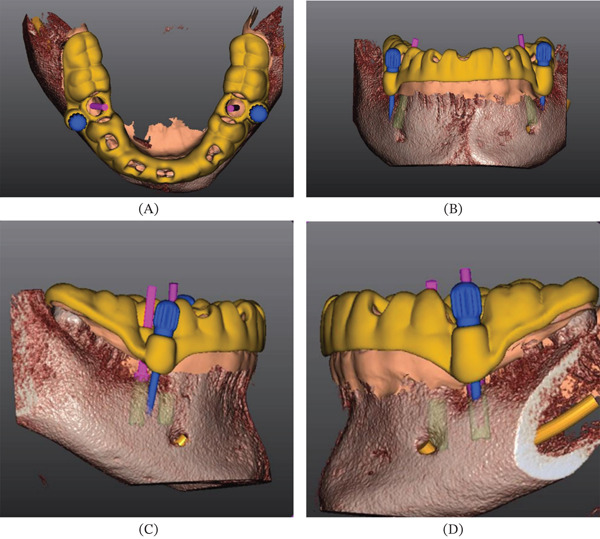
Design of the surgical guide. (A) Occlusal view with the sleeves for the implant and stabilizing screws indicated, (B) buccal view, (C) lateral view—right side, and (D) lateral view—left side.

**Figure 6:**
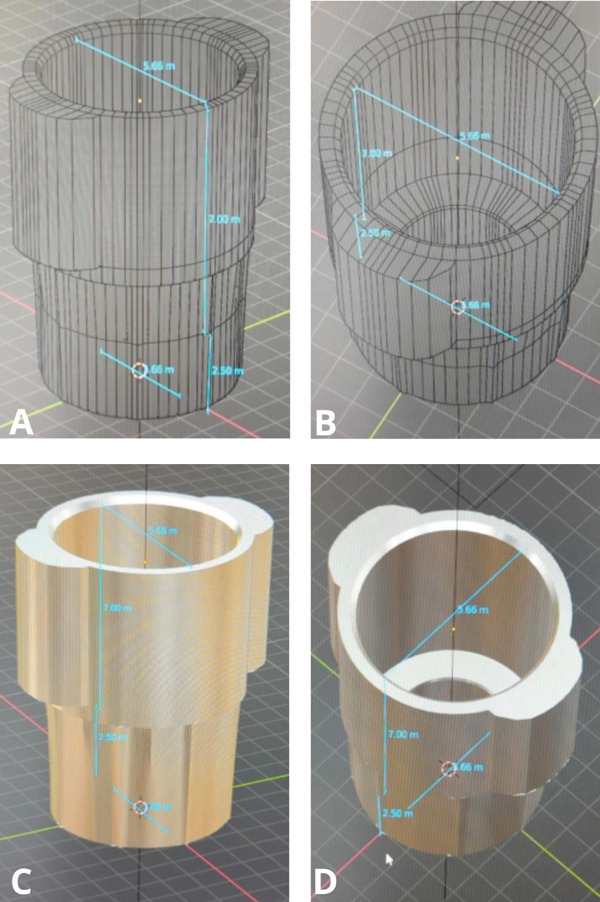
Technical development of the proprietary guiding sleeve for trephine drill navigation. (A) Design model in the CAD environment—front view, (B) design model in the CAD environment—top view, (C) visualization of the guiding sleeve—front view, and (D) visualization of the guiding sleeve—top view.

The designed surgical guide is presented in Figure 5A–D, whereas the proprietary sleeve design is shown in Figure 6A–D.

**Figure 7:**
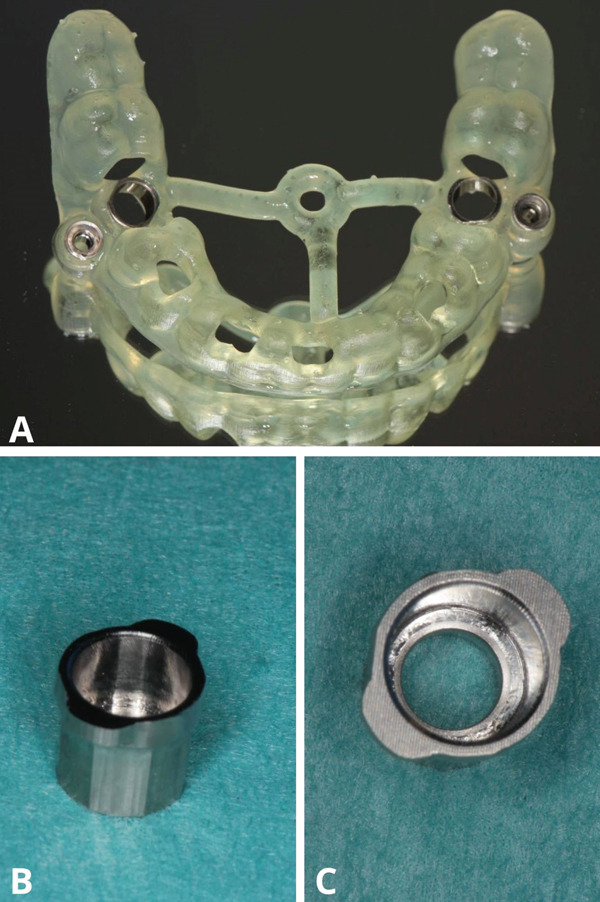
Surgical guide and guiding sleeves. (A) Surgical guide, (B) guiding sleeve—lateral view, and (C) guiding sleeve—top view.

The surgical guide was further reinforced with a stabilizing beam terminating in a ring that allows stabilization using a surgical suction device (Figure 7A–C). The appropriately selected internal diameter of the ring enables secure positioning of the guide during the procedure. The T‐shaped configuration increases the stability of the entire structure during drilling from different directions.

**Figure 8:**
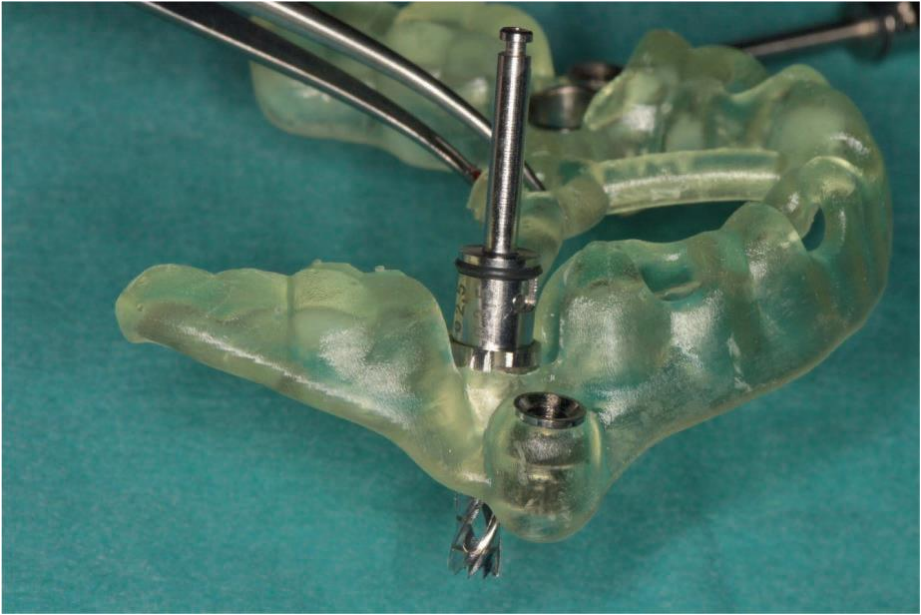
Trephine drill inserted through the dedicated guiding sleeve in the surgical guide.

The size of the designed guiding sleeve was selected to match the diameter of the trephine drill, ensuring stable guidance during the procedure (Figure 8).

**Figure 9:**
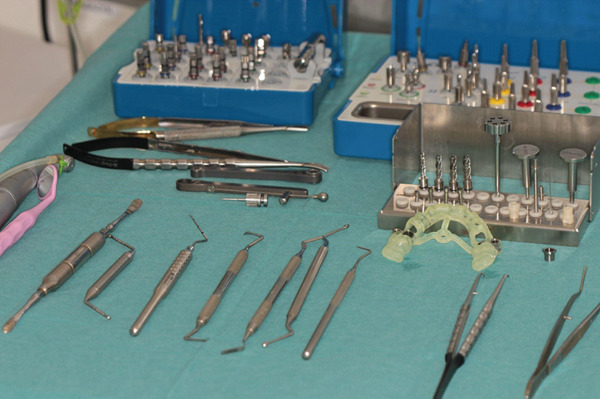
Surgical instruments prepared for the procedure, including the MGUIDE system, a set of trephines for harvesting bone cylinders according to the Khoury technique (Meisinger, Germany), the surgical guide, and standard surgical instruments.

Following completion of the planning stage and preparation of the surgical guide, the surgical procedure was performed (Figure 9).

**Figure 10:**
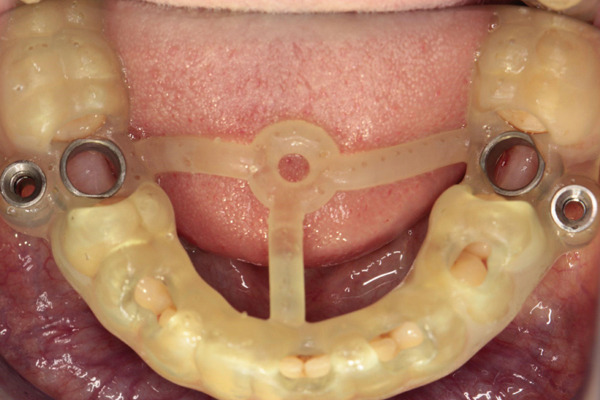
Trial placement of the surgical guide—view of the guide positioned and stabilized on the patient′s teeth.

Before the surgical procedure, a trial placement of the surgical guide was performed in the oral cavity to verify its fit and stabilization on the patient′s teeth (Figure 10). After confirming the proper seating of the guide, the subsequent stages of the surgical procedure were carried out.

**Figure 11:**
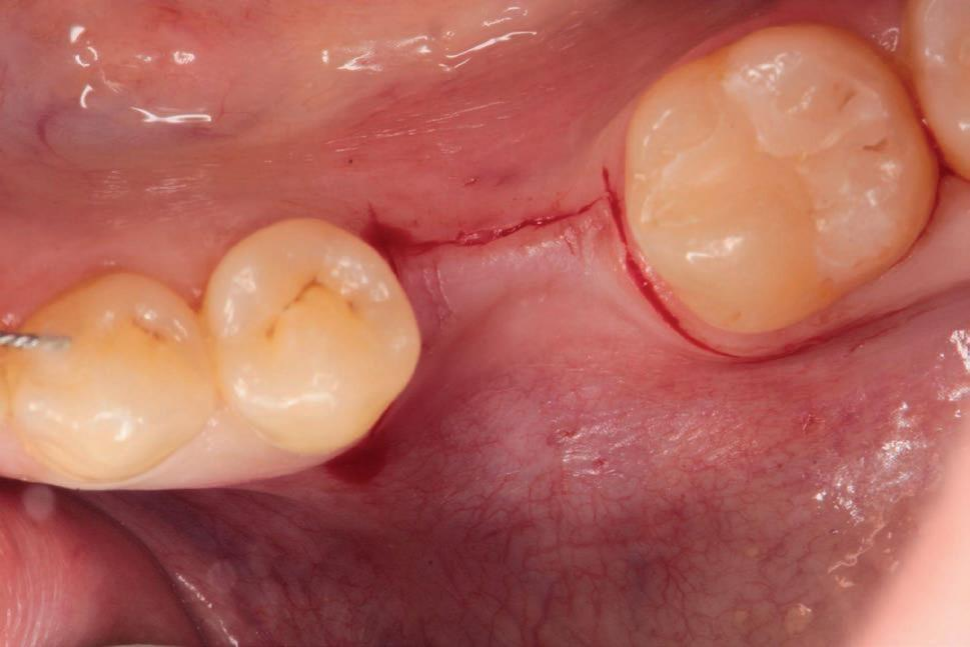
Flap incision in the region of Tooth 36 performed after marking the implant position with the surgical guide.

In the first stage of the procedure, a full‐thickness flap was performed in the region of Tooth 36 after previously marking the implant position using the surgical guide. A slight indentation created by the drill used earlier in the flapless technique was visible, indicating the planned axis of implant placement (Figure 11).

The incision was performed with preservation of the interdental papillae. Precise identification of the future implant position represents one of the key stages of the entire procedure, as it enables appropriate displacement of the soft tissues in the buccal direction, increasing the amount of keratinized gingiva and allowing the achievement of an optimal emergence profile of the future prosthetic crown without the need for additional soft tissue grafting.

**Figure 12:**
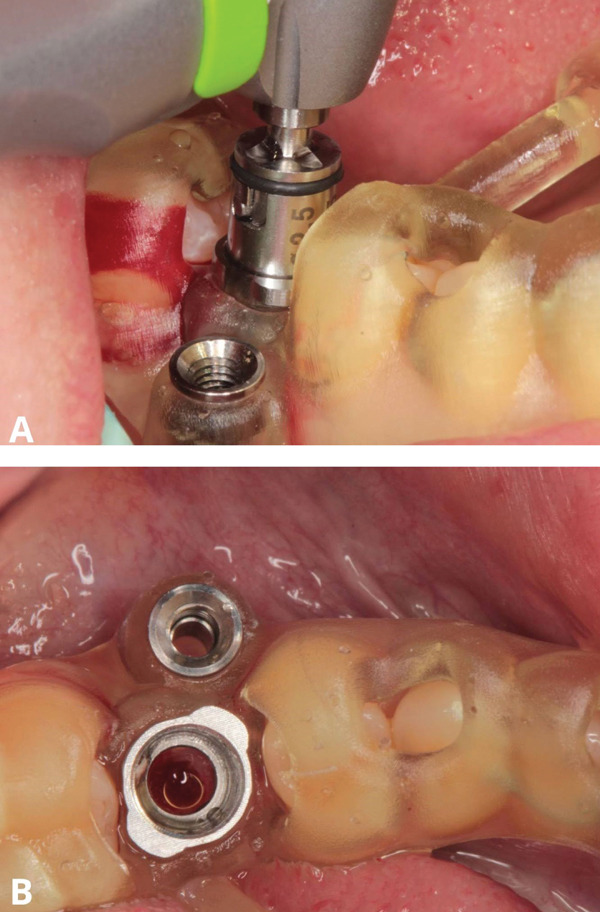
Implant site preparation using a trephine guided through the dedicated sleeve. (A) Insertion of the trephine through the guiding sleeve. (B) Surgical guide after completion of the preparation.

In contrast to the standard MGUIDE protocol, in which implant site preparation begins with a pilot drill, in the present case the first stage of preparation was performed using a trephine guided through the proprietary sleeve (Figure 12A,B). The trephine had an outer diameter of 3.5 mm and an inner diameter of 2.5 mm, and the bone core was harvested to a depth of 8 mm.

**Figure 13:**
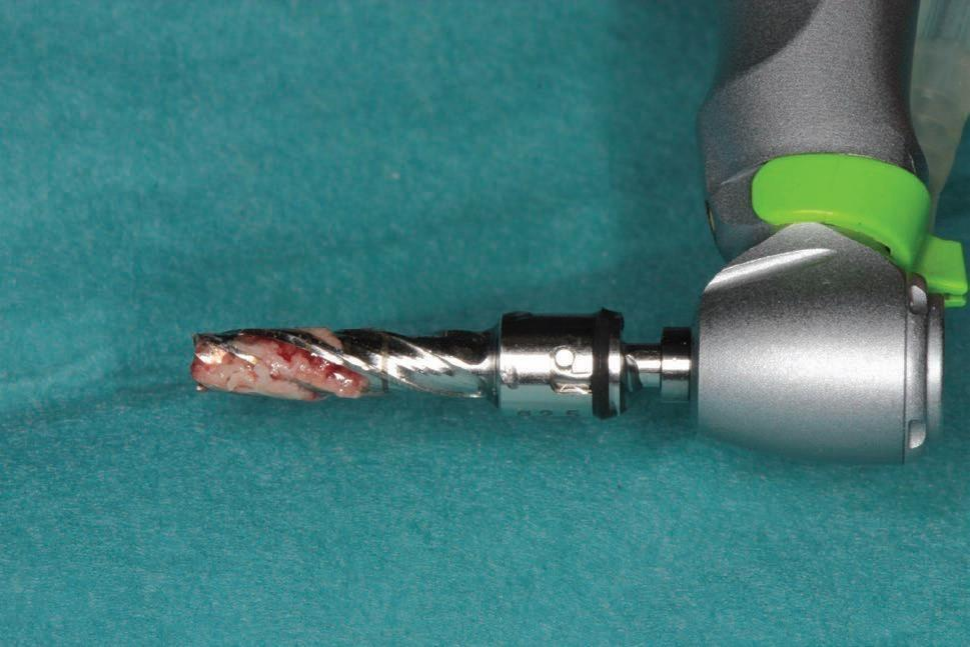
Autogenous bone cylinder harvested with a trephine during implant site preparation using the Khoury technique (Meisinger, Germany).

**Figure 14:**
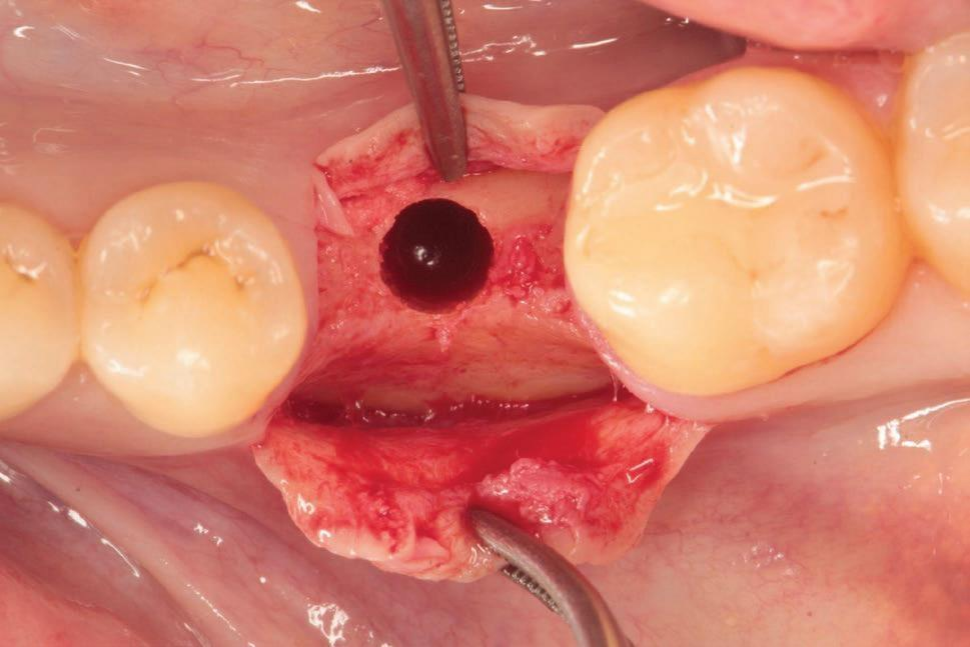
Implant site preparation at Site 36.

**Figure 15:**
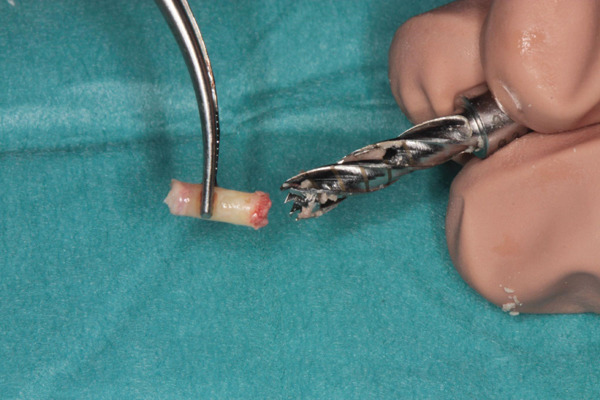
Autogenous bone graft material used for augmentation.

After preparation with the trephine, the outer part of the instrument was unscrewed and the bone cylinder corresponding to the planned diameter was gently removed (Figure 13). This allowed simultaneous initial preparation of the implant site (Figure 14) and harvesting of autogenous bone material for augmentation (Figure 15). By limiting the trephine preparation to this depth, the apical portion of the implant bed could subsequently be prepared with the dedicated implant drills, thereby maintaining the planned conical osteotomy design. This protocol was intended to reduce the risk of thermal injury in the deeper part of the osteotomy, support adequate primary implant stability, and minimize potential apical deviation.

**Figure 16:**
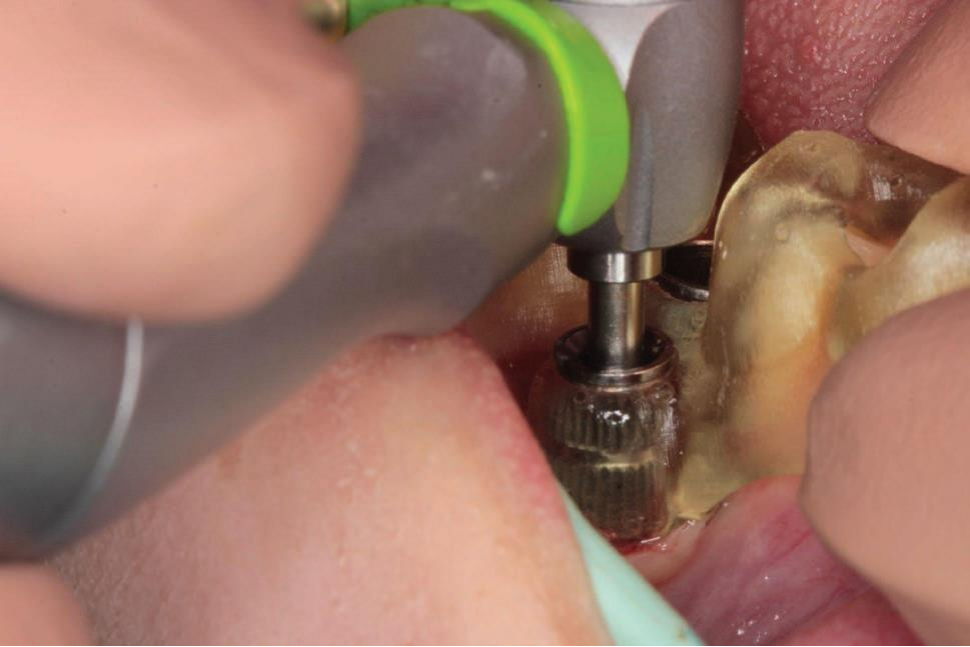
Preparation of the osteotomy for the screw stabilizing the bone graft using a pinning drill through the surgical guide.

**Figure 17:**
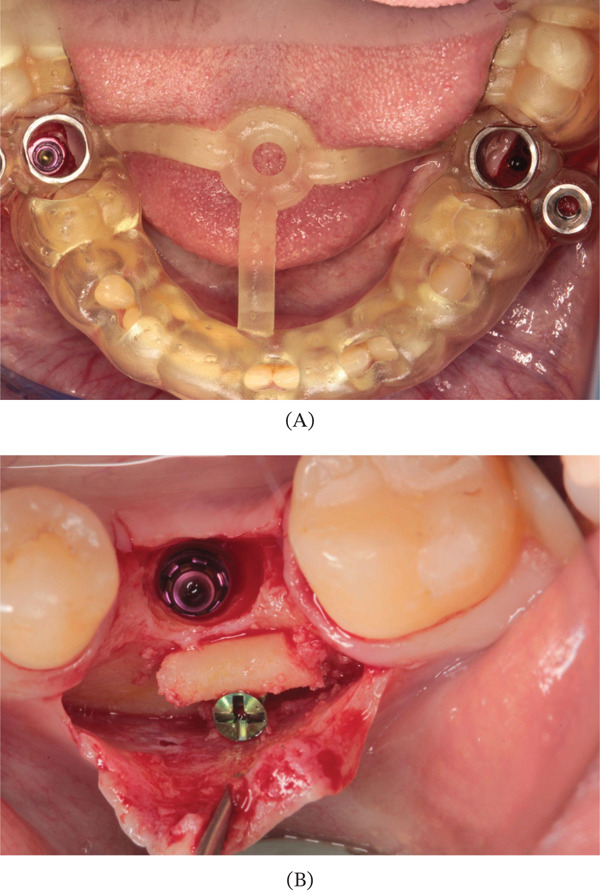
Stabilization of the bone graft at Site 36. (A) Intraoperative view after implant placement through the surgical guide. (B) Bone core stabilized with a pinning screw.

Next, a hole for the screw stabilizing the bone graft was prepared using a pinning drill (Figure 16). The implant (size 4.2 × 11.5) was placed with a final insertion torque of 40 Ncm. After implant placement through the surgical guide, the grafted bone core was stabilized with a screw, enabling precise adaptation and restoration of the deficient bone volume (Figure 17A,B).

**Figure 18:**
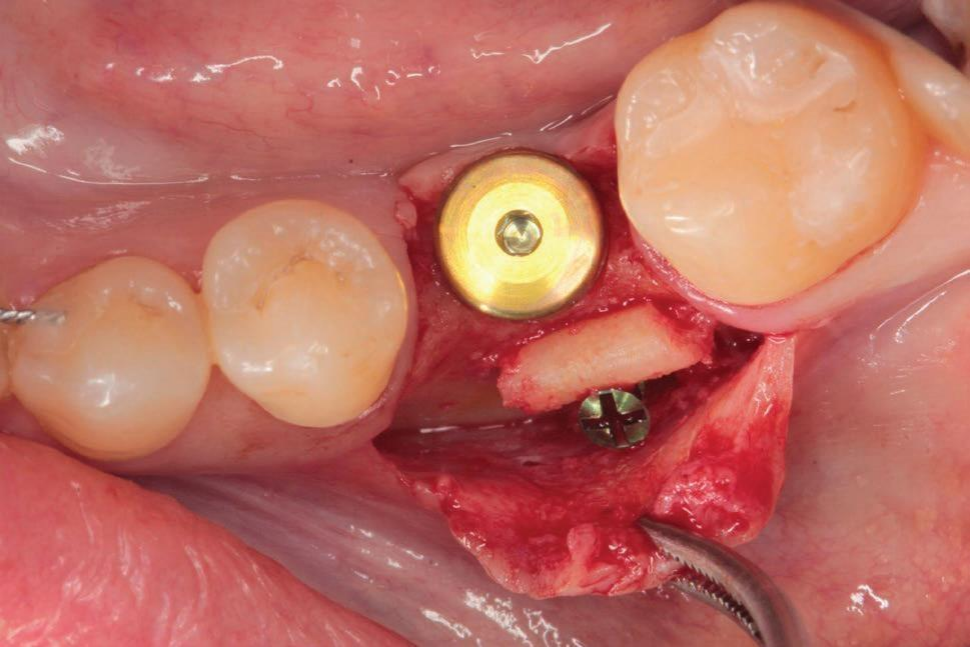
Intraoperative view after placement of the healing abutment at Site 36.

After implant placement, a healing abutment was placed. The abutment gently compressed the lingual part of the grafted bone core, stabilizing its position. The correct positioning of the implant relative to the adjacent teeth and soft tissues is visible (Figure 18).

**Figure 19:**
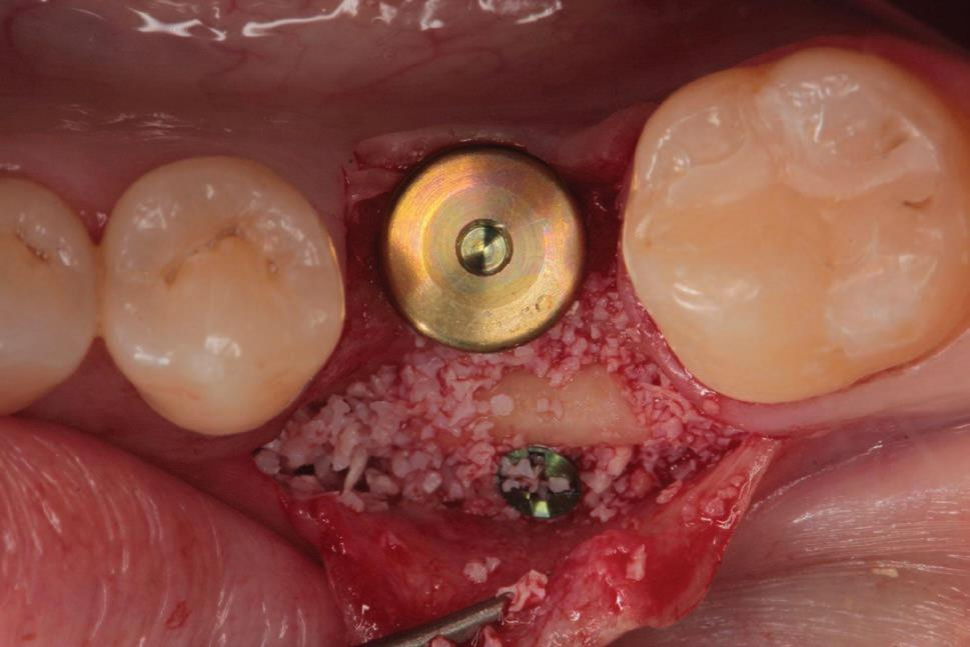
Intraoperative view after placement of the bone substitute material at Site 36.

The space between the bone core and the patient′s native bone was filled with a bone substitute material, Geistlich Bio‐Oss granules, size “L” (large granules; particle size 1–2 mm) (Geistlich Pharma AG, Wolhusen, Switzerland) (Figure 19), which allowed optimization of the augmentation contour and ensured precise adaptation of the graft to the surrounding bone. No barrier membrane was used, in accordance with Khoury′s autogenous bone‐core grafting concept [11]. The entire augmented area was covered by tension‐free soft tissue closure.

**Figure 20:**
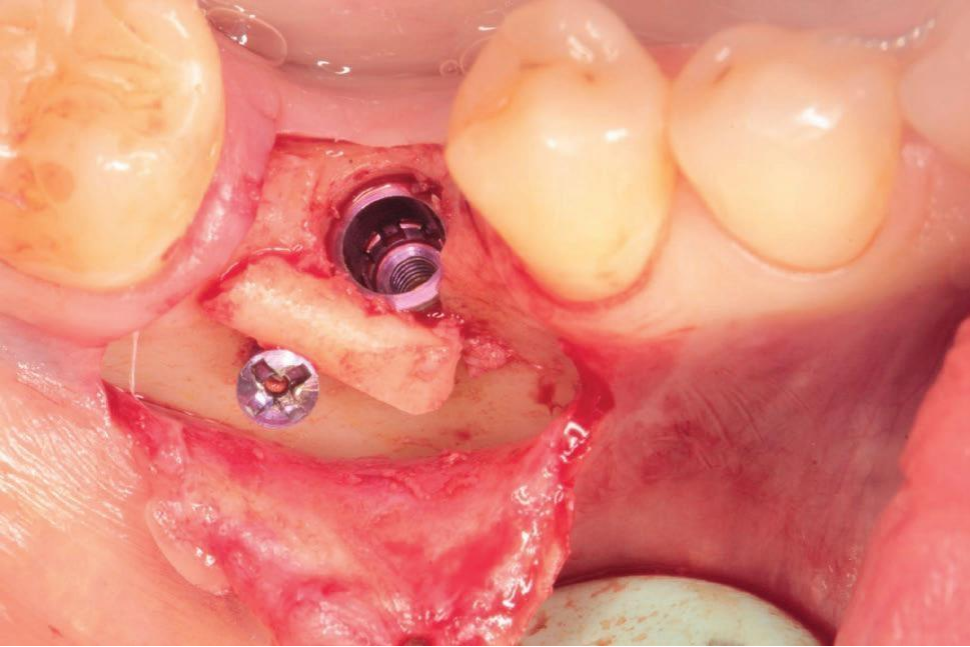
Intraoperative view after placement of the healing abutment at Site 46.

**Figure 21:**
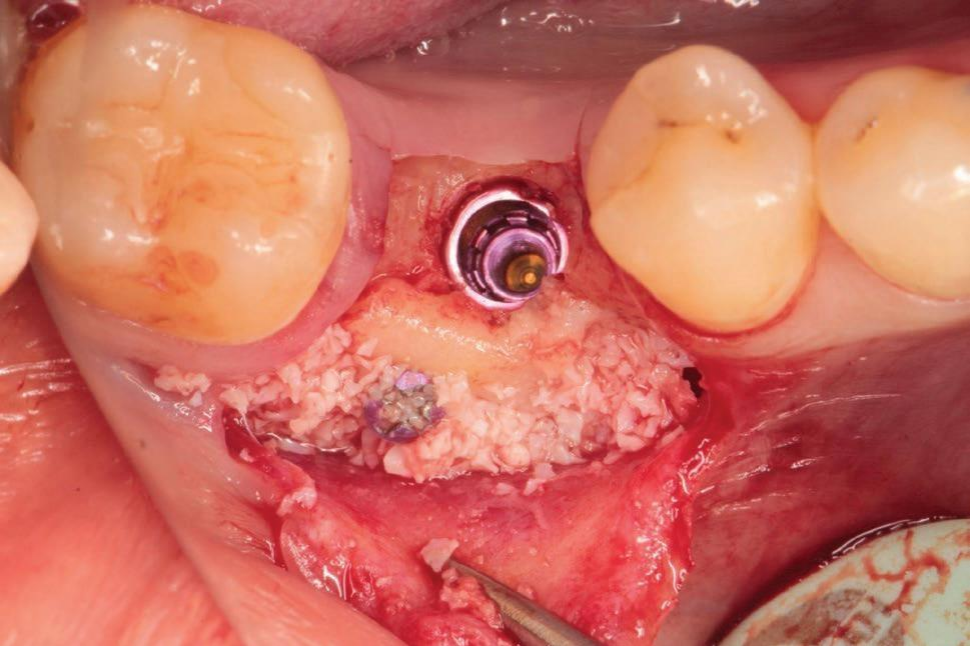
Intraoperative view after placement of the bone substitute material at Site 46.

An identical procedure was performed on the contralateral side in the region of Tooth 46, including implant placement (3.75 × 11.5; final insertion torque 40 Ncm), displacement of the soft tissues from the lingual to the buccal side, stabilization of the bone core, and augmentation with bone substitute material (Figures 20 and 21).

**Figure 22:**
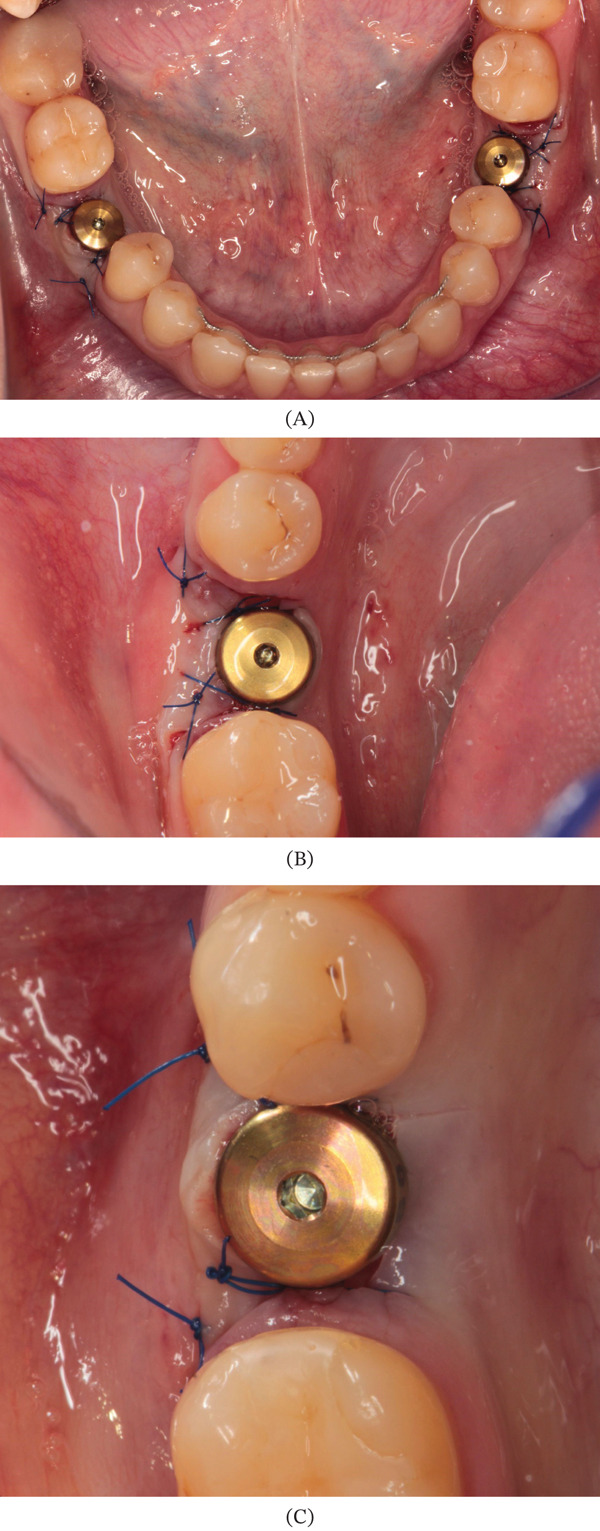
Immediate postoperative condition in the regions of Sites 36 and 46. (A) Occlusal view after suturing and placement of the healing abutments. (B) Implant Site 36. (C) Implant Site 46.

In both sites, 36 and 46, the wounds were closed with tension‐free 5‐0 sutures after placement of the healing abutments. The healing abutments additionally supported the soft tissues, allowing proper gingival shaping and formation of the future emergence profile (Figure 22A–C).

Postoperatively, a standard postoperative regimen was prescribed, including antibiotic therapy, an antiseptic mouth rinse, and nonsteroidal anti‐inflammatory drugs if required for pain control. The patient was followed clinically, and the 3‐month healing period was uneventful.

After a 3‐month healing period, the prosthetic phase of treatment was initiated. In the first step, a digital impression was obtained using dedicated scan posts from the MIS Implants Technologies system with the TRIOS intraoral scanner (3Shape).

**Figure 23:**
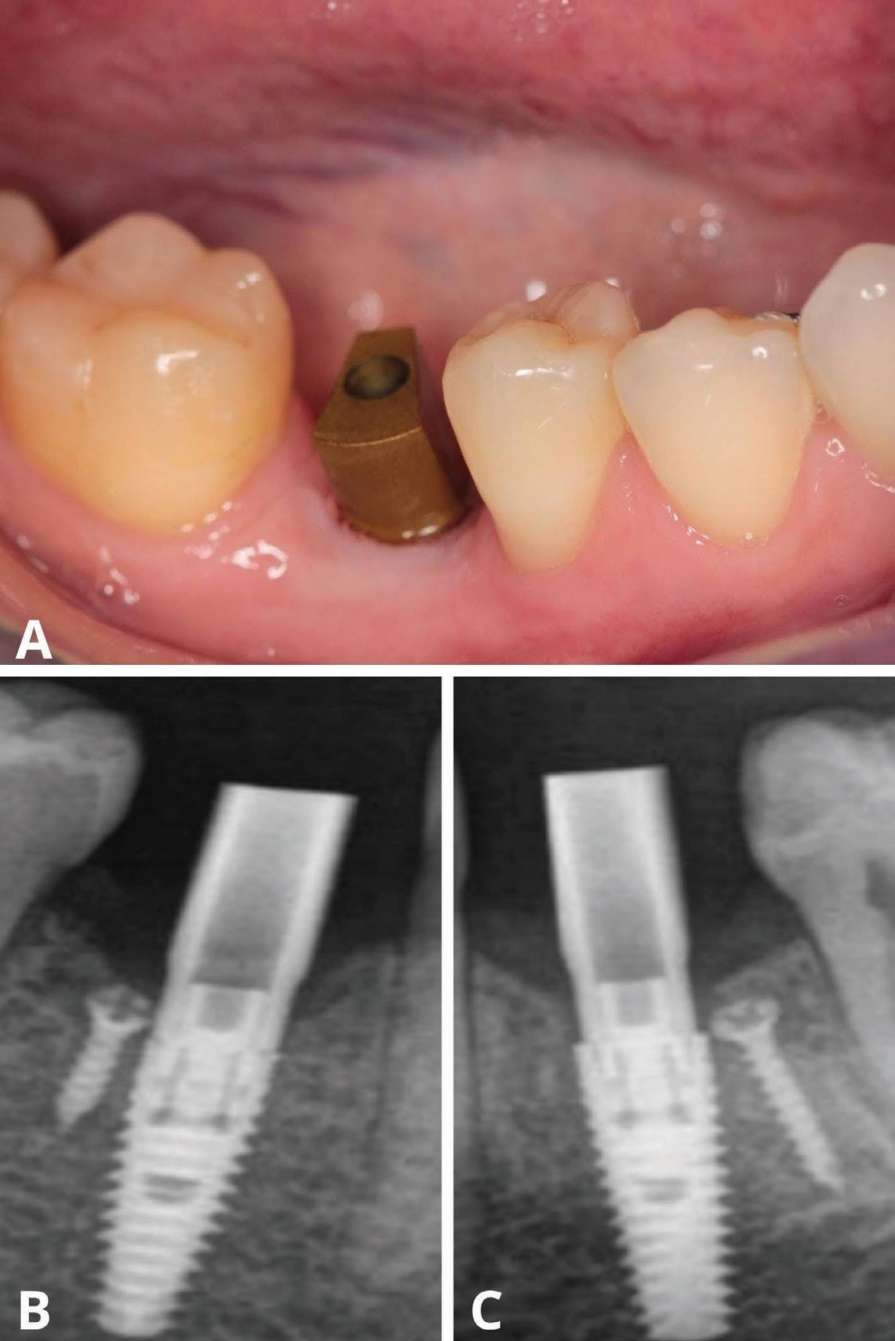
Digital impression for implant‐supported crowns. (A) Scan postscrewed onto the implant, (B) control radiograph—36, and (C) control radiograph—46.

After placement of the scan posts, a control radiograph was obtained to verify their proper fit on the implants (Figure 23A–C). The radiographic image demonstrated an appropriate level of bone reconstruction around the implants and the presence of the screws stabilizing the bone graft.

The accuracy of implant placement was assessed by comparing the planned and achieved implant positions. For the implant in Position 36, the angular deviation was 3.1°, with an apical deviation of 0.2 mm and a platform deviation of 0.4 mm. For the implant in Position 46, the angular deviation was 2.9°, with an apical deviation of 1.1 mm and a platform deviation of 0.1 mm. These values confirmed clinically acceptable transfer accuracy of the digitally guided workflow, which is particularly important in trephine harvesting because of its lower tolerance for positional deviation compared with conventional implant osteotomy.

Subsequently, screw‐retained single crowns were designed in the Exocad software.

Full‐contour zirconia was selected as the restorative material due to its high biocompatibility with soft tissues. The crowns were designed as screw‐retained implant‐supported restorations using Ti‐base abutments, thereby eliminating the risk of residual cement around the implant, which may contribute to the development of peri‐implantitis in cement‐retained prostheses [17].

The screw access channel was located on the occlusal surface of the crowns in accordance with the prosthetic design.

**Figure 24:**
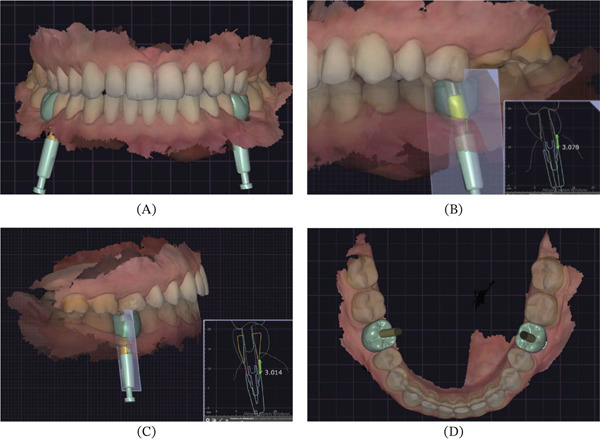
Design of the prosthetic crowns. (A) Frontal view, (B) lateral views—left side, (C) lateral views—right side, and (D) occlusal view.

Precise implant placement according to the digital plan enabled restoration of adequate hard and soft tissue volume in both the vertical and horizontal dimensions. This is confirmed by the analyses of the prosthetic designs presented below (Figure 24A–D), where the height from the implant platform to the base of the future crown measured 3.078 and 3.014 mm, respectively.

The crowns were designed with a carefully polished transmucosal surface. According to the concept described by Linkevičius et al. [18], a smooth surface in this region promotes the stability of the soft tissues surrounding the implant and reduces bacterial plaque accumulation, as the absence of surface roughness decreases bacterial adhesion [19, 20].

**Figure 25:**
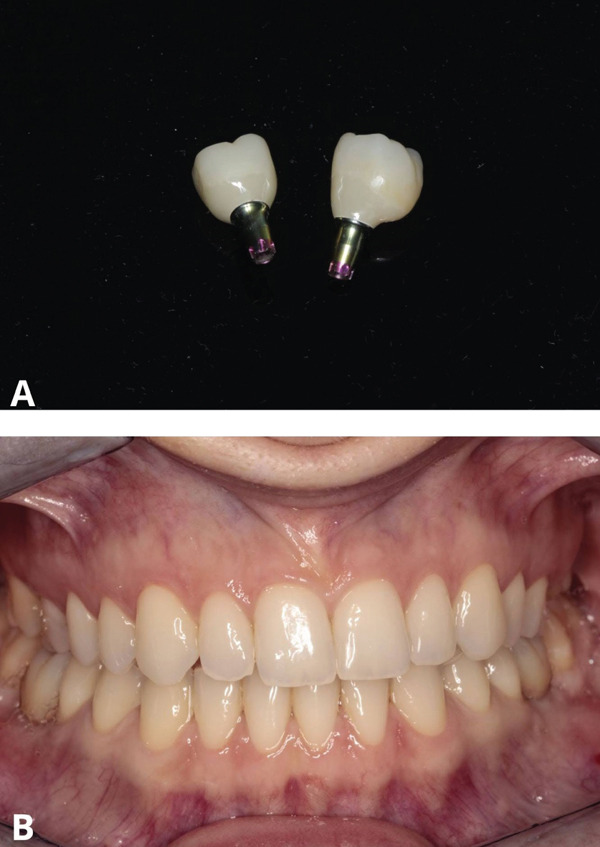
Delivery of the prosthetic restoration. (A) Implant‐supported crowns before placement. (B) Frontal view in occlusion after placement.

The crowns were placed and screw‐retained on the implants. Intraoral photographs taken immediately after their insertion show well‐formed emergence profiles and gingival tissues that were already properly shaped at the time of prosthetic delivery (Figure 25A,B).

**Figure 26:**
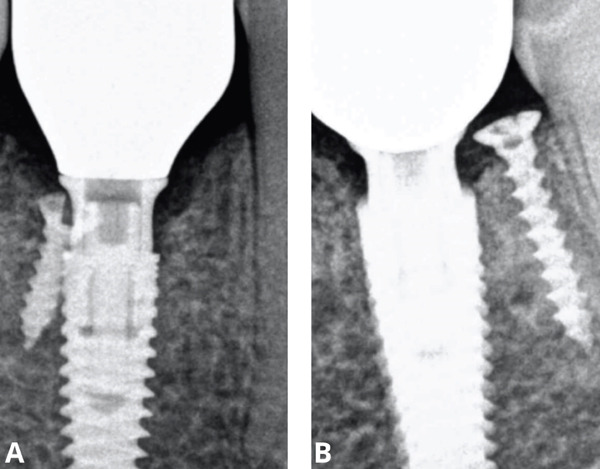
Radiographic evaluation: (A) Implant 36. (B) Implant 46.

Control radiographs confirmed an appropriate peri‐implant bone level (Figure 26A,B). The screws stabilizing the bone cores were planned to be removed at the 1‐month follow‐up visit.

**Figure 27:**
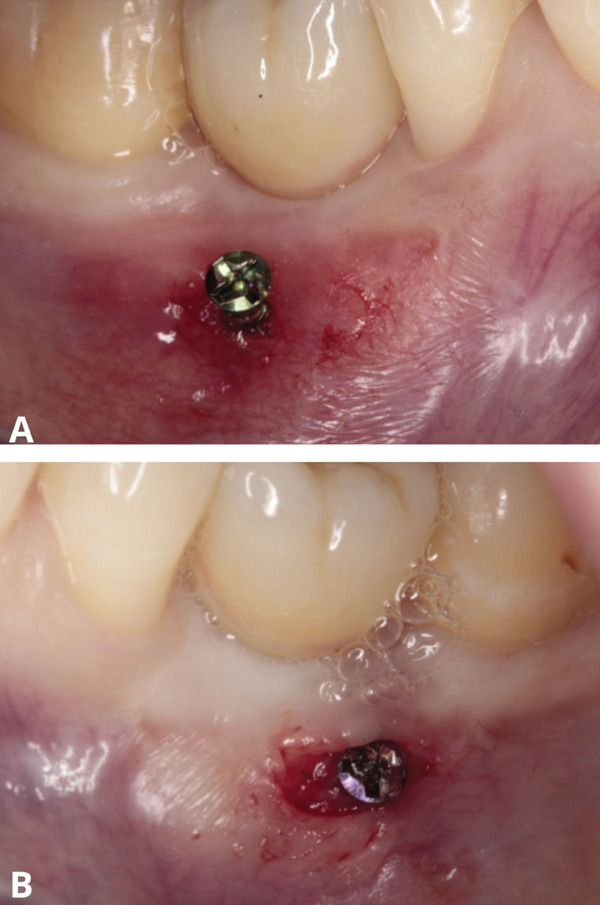
Exposure and removal of the bone graft stabilizing screw. The minimal extent of the mucosal incision allowed atraumatic removal of the screws. (A) Right side and (B) left side.

At the follow‐up visit, a small mucosal incision was performed to expose the stabilizing screws (15C scalpel; cross‐shaped incision approximately 2 × 2 mm). The characteristic metallic color of the screws (bright green and violet) facilitated their localization due to their visibility through the mucosa. This allowed precise identification and removal of the screws with minimal soft tissue incision, enabling the procedure to be performed in a minimally invasive and atraumatic manner (Figure 27A,B).

**Figure 28:**
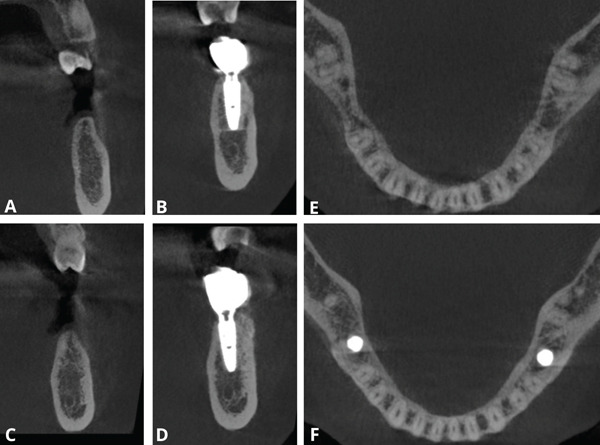
Comparative CBCT examination before and after implant treatment showing bone reconstruction at the augmentation site and correct implant positioning. (A) Cross‐sectional view before—Site 1. (B) Cross‐sectional view after—Site 1. (C) Cross‐sectional view before—Site 2. (D) Cross‐sectional view after—Site 2. (E) Axial views—before. (F) Axial views—before.

A comparative CBCT examination was performed at the site of augmentation and implant placement. The images demonstrated proper reconstruction of the augmented area and complete bone remodeling within this segment of the alveolar ridge. Accurate implant positioning consistent with the digital treatment plan was also observed (Figure 28A–D).

**Figure 29:**
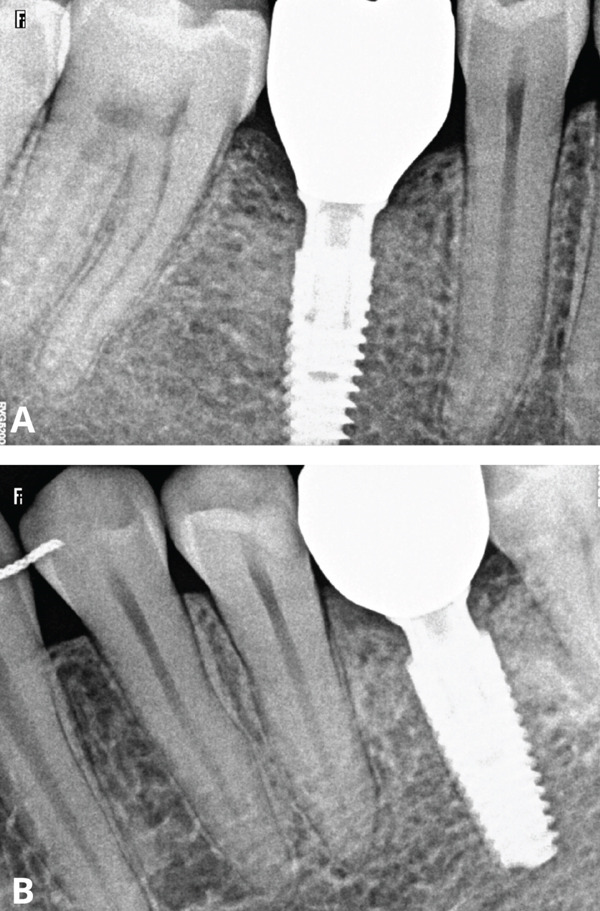
Follow‐up radiographs of implants after 3 years. (A) Site 36 and (B) Site 46.

**Figure 30:**
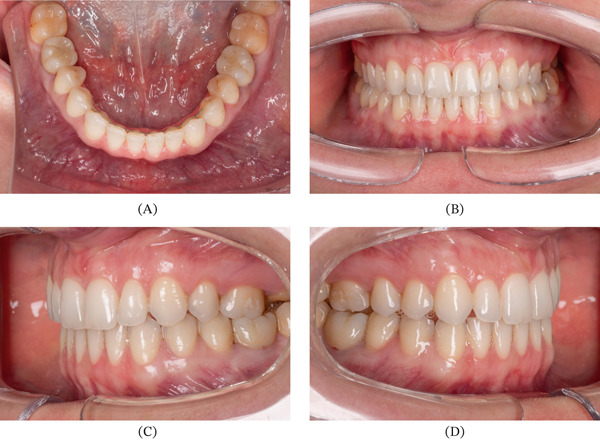
Clinical outcome 3 years after completion of treatment, demonstrating stable soft tissues and satisfactory function and aesthetics of the prosthetic restoration. (A) Occlusal view, (B) frontal view, (C) right lateral view, and (D) left lateral view.

The patient remains under regular follow‐up and professional hygiene maintenance. A 3‐year follow‐up confirmed stable hard‐ and soft‐tissue conditions based on clinical and radiographic evaluation. The results are presented in the figures below (Figures 29A,B and 30A–D).

To evaluate changes in the width of keratinized mucosa, intraoral scans obtained before surgery and after the healing period were superimposed and analyzed. In the region of Tooth 36, the width of keratinized mucosa increased from 4.549 mm preoperatively to 9.255 mm after healing, approximately 3 months before delivery of the definitive prosthetic restoration. In the region of Tooth 46, the corresponding values increased from 3.564 mm preoperatively to 7.510 mm after healing.

Cross‐sectional CBCT measurements also demonstrated an increase in bone width. In the region of Tooth 36, bone width increased from 6.67 mm preoperatively to 8.77 mm at the 1‐year follow‐up, corresponding to a gain of 2.10 mm. In the region of Tooth 46, bone width increased from 6.78 mm preoperatively to 9.75 mm at the 1‐year follow‐up, corresponding to a gain of 2.97 mm.

During further follow‐up, an additional increase in the width of peri‐implant soft tissues was observed: 1.46 mm in the region of Tooth 36 and 1.22 mm in the region of Tooth 46. These values were measured clinically with a periodontal probe during the follow‐up visit. This additional gain may be attributed to soft‐tissue adaptation to the polished submucosal surface of the monolithic zirconia crowns, as well as to the larger emergence profile and surface area of the definitive restorations compared with the healing abutments.

At the follow‐up examination, no bleeding on probing was observed at any surface around the implants in the regions of 36 and 46. The peri‐implant probing depth was 2 mm, indicating stable and healthy peri‐implant soft‐tissue conditions.

## 3. Discussion

In recent years, implant therapy based on digital planning and the use of surgical guides has become a standard approach in oral surgery. One of the key advantages of surgical guide use is that it allows precise determination of the implant position in relation to the planned prosthetic restoration, thereby enabling optimal prosthetic implant placement, increasing treatment predictability, and improving control over the surgical procedure. Shen et al. [21] assessed the accuracy of implant placement based on preoperative CAD planning performed with or without a surgical guide template. They reported that both approaches achieved satisfactory clinical outcomes; however, the use of a surgical template resulted in significantly smaller deviations between the planned and actual implant positions. The authors further suggested that surgical templates may be particularly useful in complex cases, including flapless procedures, immediate loading, aesthetic restorations, and situations with insufficient bone height.

One current limitation of digital implant planning is its relatively limited application in single‐tooth augmentation procedures. Digital software is widely used for the design of titanium meshes [22, 23] and for planning extensive bone reconstructions [24]; however, its application in small‐scale augmentations using autogenous bone cylinders remains limited.

Various surgical concepts have been proposed for horizontal and vertical alveolar ridge augmentation, including osteotomy‐based procedures, distraction osteogenesis, particulate guided bone regeneration, and block grafting techniques. Osteotomy techniques, such as ridge splitting or sandwich osteotomies, preserve crestal soft tissues, attached gingiva, and, in selected cases, papillae; however, they are technique‐sensitive and carry a risk of fracture within the treated segment. Distraction osteogenesis enables gradual bone formation through controlled segment movement, but its routine use in implant dentistry is limited by device‐related costs, patient discomfort, and the need for sufficient residual bone volume. Particulate guided bone regeneration relies on barrier membranes to stabilize the graft, exclude soft tissue ingrowth, and reduce resorption, although complex vertical defects often require titanium‐reinforced or nonresorbable membranes, which may be associated with membrane exposure and the need for a second surgical procedure. Block augmentation, particularly with autogenous bone, remains a well‐established option because of its mechanical stability and lower resorption compared with particulate grafts, but it requires precise adaptation, fixation, and careful soft tissue management [25].

Against this background, the Digital CarroTrack Technique may be regarded as a digitally guided modification of autogenous block augmentation using a cylindrical bone graft. In the presented case, an individually designed guiding sleeve for the trephine drill was used, allowing precise harvesting of the bone cylinder and its guided placement from the buccal side. This approach was termed the Digital CarroTrack Technique and represents a digital modification of the autogenous bone cylinder harvesting technique, or “carrot technique,” described by Khoury and Doliveux [11]. It allows digital implant planning to be integrated with an augmentation procedure using autogenous bone material.

The applied method offers several potential clinical advantages. These include the possibility of performing the procedure within a single surgical field, reducing the need for bone harvesting from a separate donor site, increasing procedural precision, and potentially shortening the postoperative recovery period.

**Figure 31:**
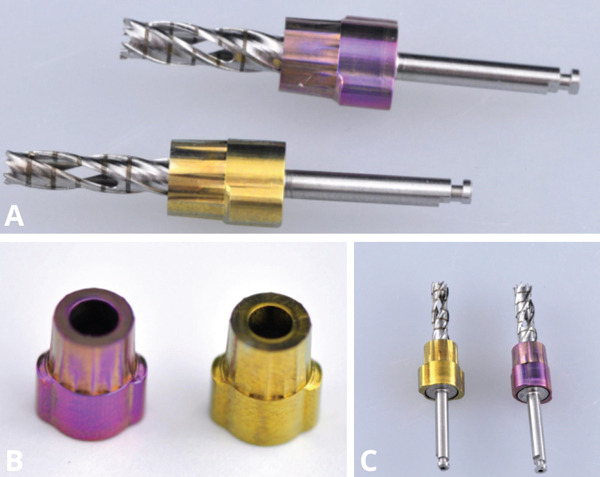
(A–C) Guiding sleeves designed for various trephine drill diameters used in the navigated bone cylinder harvesting technique (authors′ proprietary design). (A) Trephine drills with guiding sleeves—side view, (B) guiding sleeves, (C) trephine drills with guiding sleeves—top view.

In a further stage of development of the presented solution, guiding sleeves dedicated to different diameters of trephine drills were designed, allowing even more precise adjustment of the harvested bone cylinder width to the alveolar ridge width and the extent of the required augmentation (Figure 31A–C).

However, it should be emphasized that the presented method requires further clinical studies, including comparative studies and statistical analyses, to evaluate its effectiveness, predictability, and potential limitations in clinical practice.

## 4. Patient Perspective

The patient reported satisfaction with the treatment outcome, improved masticatory function, and overall comfort after the completion of treatment.

## 5. Informed Consent

Formal ethical approval was not required for this study as it is a single anonymized case report. Written informed consent was obtained from the patient for the publication of this case report and the accompanying clinical and radiological images.

## Author Contributions

Conceptualization: Dr. Jakub Kwiatek, and M.Sc. Marta Leśna; methodology: Dr. Jakub Kwiatek, M.Sc. Marta Leśna, and Sylwia Pokorska, dentist; software: M.Sc. Marta Leśna, Sylwia Pokorska, dentist, and Noemi Konopelski, dentist; validation: Dr. Jakub Kwiatek and M.Sc. Marta Leśna; formal analysis: M.Sc. Marta Leśna, Dr. Jakub Kwiatek, and Oskar Barczak, dentist; investigation: Dr. Jakub Kwiatek, M.Sc. Marta Leśna, and Sylwia Pokorska, dentist; resources: Dr. Jakub Kwiatek; data curation: Marcin Lenkowski, dentist, M.Sc. Marta Leśna, Oskar Barczak, dentist, and Ilona Różewicz, dentist; writing—original draft preparation: M.Sc. Marta Leśna, Sylwia Pokorska, dentist, and Paulina Łojewska‐Pabiś, dentist; writing—review and editing: Dr. Jakub Kwiatek; visualization: M.Sc. Marta Leśna, Noemi Konopelski, dentist, and Oskar Barczak, dentist; supervision: Dr. Jakub Kwiatek; project administration: Dr. Jakub Kwiatek and M.Sc. Marta Leśna.

## Funding

No funding was received for this manuscript.

## Disclosure

All authors have read and approved the final version of the manuscript. The corresponding author had full access to all of the data in this study and takes complete responsibility for the integrity of the data and the accuracy of the data analysis.

## Conflicts of Interest

Author Jakub Kwiatek is the inventor of the guiding sleeves described in this study, which are subject to patent application PL 449269. All other authors declare no conflicts of interest.

## Data Availability

The data that support the findings of this study are available from the corresponding author upon reasonable request.
